# Salicilic Acid as a Prophylactic

**Published:** 1882-01

**Authors:** 


### SALICILIC ACID AS A PROPHYLACTIC.

This acid enjoys a large reputation as a preventive. In small quantities added to saccharine liquids it prevents fermentation. Combined in minute proportions with delicate alkaloid solutions, it preserves them from taking on those peculiar cryptogamic growths common to certain aqueous solutions, and in a variety of ways it has become a useful and efficient physiological agent. But in a late experience it was proven that its antiseptic action cannot be depended upon when combined with any liquid substance in vessels of wood. It is claimed that the acid under these circumstances quickly disappears, apparently being decomposed and absorbed by the tissue of the wood. To guard against this trouble, it is recommended to coat the vessel with pitch before putting it to the use intended.

